# Mental Well‐Being Differences in Cohabitation and Marriage: The Role of Childhood Selection

**DOI:** 10.1111/jomf.12431

**Published:** 2017-07-24

**Authors:** Brienna Perelli‐Harris, Marta Styrc

**Affiliations:** ^1^ University of Southampton

**Keywords:** childhood, cohabitation, marriage, mental health

## Abstract

Prior studies have found that marriage benefits well‐being, but cohabitation may provide similar benefits. An analysis of the British Cohort Study 1970, a prospective survey following respondents to age 42, examines whether partnerships in general, and marriage in particular, influence mental well‐being in midlife. Propensity score matching indicates whether childhood characteristics are a sufficient source of selection to eliminate differences in well‐being between those living with and without a partner and those cohabitating and married. The results indicate that matching on childhood characteristics does not eliminate advantages to living with a partner; however, matching eliminates differences between marriage and cohabitation for men and women more likely to marry. On the other hand, marriage may provide benefits to women less likely to marry unless they have shared children and are in long‐lasting partnerships. Hence, childhood selection attenuates differences between cohabitation and marriage, except for women less likely to marry.

Numerous studies have found that marriage benefits mental well‐being (for a review, see Waite & Gallagher, 2000; Lamb, Lee, & DeMaris, 2003; Simon, 2002). The strength and persistence of these findings have led some policy makers to call for programs that encourage marriage. For example, pro‐marriage policy initiatives were pushed during the George W. Bush administration in the United States (Bir et al., 2012), and the current conservative UK government has legislated tax breaks for married couples (BBC News, 2015). Much of the research underlying these initiatives, however, has compared the married and unmarried, without distinguishing between those who were cohabiting or single (e.g., Hughes & Waite, 2009; Liu & Umberson, 2008; Waite & Gallagher, 2000). Some studies have begun to examine differences between cohabitation and marriage with respect to mental health and depressive symptoms (e.g., Brown, 2000; Lamb et al., 2003; Musick & Bumpass, 2012), but most of these studies use American cross‐sectional data and may not sufficiently control for selection effects. Less is known about the relationship between marriage and mental health in other contexts, where cohabitation is widespread. Given the recent increase in cohabitation and its changing meaning (Berrington, Perelli‐Harris, & Trevena, 2015; Perelli‐Harris et al., 2014), it is important to revisit whether partnerships in general, and marriage in particular, continue to provide distinct benefits to well‐being, especially for those who are less likely to marry.

In this study, we examine whether being in a partnership and the type of the partnership—marriage or cohabitation—are associated with higher mental well‐being in midlife in the United Kingdom. In the United Kingdom, cohabitation has become the normative pathway to union formation: From 2004 to 2007, 80% of all marriages started with premarital cohabitation, and the duration of cohabiting unions has been steadily increasing (Beaujouan & Ni Bhrolchain, 2011). Cohabitation has also become common for childbearing: In 2012, 30% of all babies were born to cohabiting mothers (Office for National Statistics, 2013). The pervasiveness of cohabitation, especially if it takes on much of the form and function of marriage, suggests that cohabitors may have well‐being similar to that of married individuals.

Nonetheless, a large number of studies across countries have shown distinct differences between cohabitation and marriage with respect to depressive symptoms in the United States (Brown, 2000), relationship quality across Europe (Wiik, Keizer, & Lappegård, 2012), and life satisfaction in Europe (Soons & Kalmijn, 2009). Some of the studies that examined the relationship between marriage and psychological or subjective well‐being employed a panel design that starts in young adulthood, providing insights into transitions during a short period but not directly comparing long‐term cohabiting and marital unions and their effects in midlife (Germany: Zimmerman & Easterlin, 2006; the Netherlands: Soons, Liefbroer, & Kalmijn, 2009; United States: Musick & Bumpass, 2012). For example, using fixed effects models, Musick and Bumpass (2012) found that in the United States, transitions into cohabitation and marriage have similar effects on psychological, health, and social well‐being, and any differences found are relatively small. Musick and Bumpass (2012), however, did not examine the consequences of cohabitation versus marriage later in the life course, after the typical postponement of marriage throughout young adulthood and after the majority of childbearing. In addition, although this and other studies using fixed effects models examined variation within individuals over time, they did not compare across individuals with different characteristics that select people into cohabitation or marriage, particularly drawing on selection mechanisms occurring early in life, before the entrance into adulthood. These studies did not examine whether marriage was likely to increase well‐being for those who were less likely to marry, often those with disadvantaged backgrounds and targeted by pro‐marriage policies.

Our study uses propensity score matching to investigate differences between marriage and cohabitation in the United Kingdom. Although union status changes during the life course and cohabiting couples often marry (Perelli‐Harris & Lyons‐Amos, 2015), we consider marriage a “treatment,” because couples must officially decide to marry and act on that decision. Our propensity score matching (PSM) strategy examines whether people with similar background characteristics are more likely to have higher mental well‐being scores if they marry. This approach takes into account important selection characteristics that predict both union formation and well‐being, but cannot control for the respondents' current situation. By matching people with similar characteristics, however, we can ascertain whether marriage provides benefits beyond living with someone as well as the heterogeneity of treatment effects—whether the effects of marriage differ for those with a higher or lower propensity to marry based on childhood characteristics. Our data, the British Cohort Study, followed the 1970 birth cohort up to age 42. This cohort experienced partnership formation in the 1990s and 2000s, which is more recent than many previous studies (e.g., Musick & Bumpass, 2012). In addition, cohabitation in midlife is relatively understudied despite it becoming a more common phenomenon in Britain (i.e., 20% of adults in our survey were cohabiting at age 42). Given that we are not interested in the timing of marriage or cohabitation per se, the PSM approach is appropriate for examining whether currently being in a marriage matters. However, because the duration of and investments in a union may signal the positive benefits of the partnership, we also compare the type of union for those who have been in long‐lasting partnerships, never experienced union separation, and have had children together. In addition, we compare results for men and women who may experience varying effects of different types of partnerships on well‐being (Simon, 2002; Williams, 2003).

We are particularly interested in whether early life conditions attenuate the association between partnership type on later life outcomes, contributing to the growing body of research investigating these links (Elo, 2009; Kuh, Power, Blane, & Bartley, 2004; Umberson, Crosnoe, & Reczek, 2010). Early childhood factors often precondition individuals to choose cohabitation or marriage, but they are also strongly linked to mental well‐being in adulthood and may therefore reduce or eliminate the association between marriage and well‐being. Hence, this study not only provides insights into whether marriage makes a difference to well‐being beyond simply living in a partnership, it also contributes to our understanding of the role of early life conditions in understanding these differences.

## Background

### Current Relationship Status

An individual's current partnership status, regardless of whether married or cohabiting, is potentially the most relevant to current well‐being. Living in a partnership usually provides sexual and emotional intimacy, companionship, and daily interaction, which can promote well‐being. An intimate partner can provide care and social and emotional support and encourage healthy behaviors (Umberson et al., 2010). In addition, partners often link each other to greater friendship and kin networks that can provide social support (Ross, 1995). Living together and sharing a household can lead to economies of scale. The savings incurred may be particularly important for low‐income couples, who in qualitative interviews in the United Kingdom have mentioned that the decision to move in together was motivated by housing costs (Berrington et al., 2015).

Beyond simply living with a partner, however, marriage may provide unique benefits to well‐being (Waite & Gallagher, 2000). Marriage is often a social sign of commitment, or “enforceable trust” (Cherlin, 2004, p. 854). The symbolic promise of marriage may provide couples with a long‐term perspective that the future of their relationship is secure. Because marriages are legally harder to dissolve, couples may be more motivated to work through their disagreements, thereby maintaining union stability and, with it, general life stability. The long‐term perspective may also benefit personal and social controls, meaning spouses deliberately influence each other's personal behavior because they want them to be healthy and live longer (Umberson, 1987). The reduction in life uncertainty and increased care could enhance well‐being (Liu & Umberson, 2008; Soons et al., 2009) and even result in psychological or cognitive changes that promote mental well‐being (Li, Liu, & Guo, 2015). These benefits may be enhanced further through personal networks, such as in‐laws, who provide structural social support and coping resources to married couples because the relationships are more defined (Marcussen, 2005). In addition, the UK legal system continues to favor marriage in terms of inheritance tax and access to the courts when unions dissolve, which may influence the level of perceived security (Perelli‐Harris & Sanchez Gassen, 2012). Although general social disapproval of cohabitation is low in Britain, the social expectation to marry is still pervasive (Berrington et al., 2015). Thus, although living with someone may result in many of the same benefits to mental well‐being, in today's Britain, marriage may still be a sign of a more committed relationship and confer additional social and legal benefits, which would in turn enhance well‐being.

### Long‐Lasting and First Partnerships

Although current partnership status conveys certain immediate benefits, longer union duration is usually a sign of a stable, committed relationship potentially providing a greater boost to well‐being (Berrington et al., 2015; Duncan & Philips, 2008; Jamieson et al., 2002). Poor quality relationships are more likely to end, and relationships with negative effects on well‐being are dwindle. Over time, commitment increases, and couples are likely to invest more in the relationship, for example, by investing in housing or pooling resources (Heimdal & Houseknecht, 2003; Lyngstad, Noack, & Tufte, 2010). Long‐term cohabiting relationships tend to reflect deeper commitment and, given the lack of social sanctions against cohabitation in the United Kingdom (Duncan & Philips, 2008), may be no different from marriage with respect to well‐being.

On the other hand, as people adapt to marriage and cohabitation, they often return to their initial levels of well‐being (Lucas & Clark, 2006; Soons et al., 2009; Zimmerman & Easterlin, 2006). Relationship quality tends to decline over time, as partners become used to each other, and the honeymoon effect wears off. One Dutch study observed that entrance into cohabitation and marriage increased subjective well‐being, with marriage providing the highest boost to well‐being, but moderate adaptation occurred in the long run (Soons et al., 2009). Soons et al. (2009) argue, however, that the return to previous levels occurs slowly, especially when compared with the never partnered, whose well‐being declines more rapidly. Thus, union duration appears to work in contradictory ways: Unions of longer duration imply greater commitment and investment in the relationship, but at the same time, subjective well‐being tends to decline after the honeymoon period. The question is whether unions that have made it through this period are similar in their effects on well‐being, regardless of whether they are cohabiting or marital.

Partnership dissolution may also negatively influence mental well‐being (Amato, 2010; Carr & Springer, 2010). Previous research has found that divorce inflicts costs on physical and mental health for many years, even for those who remarry (Hughes & Waite, 2009). People who cohabit may be more at risk for the negative effects of union dissolution because cohabiting unions have higher dissolution rates than marriage (Beaujouan & Ni Bhrolchain, 2011). A greater proportion of those currently cohabiting may be repartnered than those currently married. In addition, people who have separated or divorced are more likely to choose cohabitation for subsequent relationships (Gałęzewska, Perelli‐Harris, & Berrington, Forthcoming), and second‐order or higher order partnerships often have higher dissolution rates and worse relationship quality (Hughes & Waite, 2009; Sweeney, 2010). Thus, because cohabitors are more likely to have experienced union dissolution, it is important to compare cohabitors and married people living with their first partner to eliminate any lingering effects of partnership instability.

### Unions With Children

Having shared children can be an important sign of investment in a relationship. Previous studies have considered childbearing to be an indicator of the similarity between cohabitation and marriage (Heuveline & Timberlake, 2004; Raley, 2001). Similar to married parents, cohabiting parents have a shared interest in their children, can provide care and other resources, and may work harder to maintain their relationship to ensure stability. Unmarried fathers in the United Kingdom have the same rights as married fathers and face little social disapproval for not marrying their child's mother (Barlow, 2014; Park & Rhead, 2013). Nonetheless, studies show that cohabiting parents continue to be different from married parents; for example, in the United Kingdom, cohabiting parents are more likely to separate (Goodman & Greaves, 2010) and have lower second birth rates than their married counterparts (Perelli‐Harris, 2014). Hence, cohabiting parents with shared children may continue to have different well‐being than married parents.

### Gender Differences in Benefits to Marriage and Cohabitation

Sociologists have long questioned whether men receive greater psychological benefits from marriage than women, and whether there is a “his” and “her” marriage (Bernard, 1982; Williams, 2003). Evidence for this argument is weak; most studies find few differences in the benefits from marriage between men and women (Robles, Slatcher, Trombello, & McGinn, 2014; Ross, 1995; Simon, 2002; Williams, 2003). These studies, however, did not directly compare cohabitation and marriage, and some subtle differences may emerge for these two types of relationships. The advantages that an intimate coresidential relationship convey—for example, sexual and emotional intimacy, pooled resources, and shared household investments—may be sufficient to provide mental health benefits to men. However, cohabitation may lack the same symbolic meaning or commitment as marriage, which may be more important for women. In British focus groups, Berrington et al. (2015) found that both men and women believed women place a greater value on a “proper” wedding, both as a symbol of commitment and as an expression of tradition, feminism, and romantic fantasy. Not experiencing the “big day,” may lead some women to feel discontent with their lives. In addition, because cohabitation does not convey the same legal protection as marriage, women may prefer the security that marriage provides, especially if they have a lower level of income or take time out of the labor force to maintain the household. Hence, men and women may value marriage differently, which may lead to differential effects by gender.

### Selection Based on Childhood Circumstances

The benefits of partnerships and marriage may not be causal, but instead the result of social selection; differences in well‐being are a result of the characteristics of the people who choose to be in a particular type of partnership. In our study, we focus on childhood characteristics that occur before the “treatment,” or entering into an adult partnership. Parental influences and characteristics that have developed in childhood are very important for determining later life outcomes (for reviews, see Elo, 2009; Kuh et al., 2004). Health and mortality research suggests that the “long arm of childhood” extends into adulthood and is a significant predictor of adult health outcomes (Hayward & Gorman, 2004; Palloni, 2006). Our research includes a range of childhood characteristics that could attenuate the distinction between cohabitation and marriage.

Parental socioeconomic status is one of the most significant predictors of future life outcomes (Case, Fertig, & Paxson, 2005; Luo & Waite, 2005). The intergenerational transmission of conditions and behaviors is extremely important for educational trajectories, social mobility, and future employment (Goodman, Joyce, & Smith, 2011), all of which can have implications for both partnerships and mental well‐being. Parents' socioeconomic position influences childhood development and adult outcomes through a complex set of transmission mechanisms, including values and attitudes, resources, behaviors, social interactions (Goodman et al., 2011), and genetic endowments (Li et al., 2015). In many contexts, parental characteristics also directly influences choices to cohabit or marry (Wiik, 2009). For example, in Britain, the mother's age at birth and the father's social class were associated with entrance into cohabitation (Berrington & Diamond, 2000). Parents' marital status and divorce in childhood can also hinder the development of interpersonal and relationship skills, cognitive growth, and educational achievement (Amato, 2010; Kim, 2011), which again may influence both partnership formation and mental health. Parental divorce often leads children to reject the institution of marriage and adopt more favorable attitudes toward cohabitation and divorce (Axinn & Thornton, 1996) as well as choose cohabitation for their own relationships (Perelli‐Harris, Gałęzewska, Sánchez Gassen, Berrington, & Holland, 2017). Thus, the beneficial effects of marriage to mental health may not be a result of marriage, but instead childhood socioeconomic status and stability, which would lead individuals to marry in adulthood.

Children's cognitive development and educational aspirations may have an effect on both future mental well‐being and partnership formation that is independent of parental socioeconomic status. Cognitive functioning in childhood is closely associated with adult positive mental health (Hatch et al., 2007), and cognitive test scores in childhood are highly predictive of future educational attainment (Feinstein & Bynner, 2004), which may also influence adult mental health. However, education is not only associated with mental health; recent studies have indicated that education is associated with marriage in most countries (Kalmijn, 2013).

An individual's psychological attributes, physical health, values, and attitudes in childhood can also have major influences on both partnership formation and mental well‐being. Psychological attributes, for example, psychological distress, self‐esteem, locus of control, and behavioral problems, are usually predictors of future mental health (Goodman et al., 2011) or even an alternative way of measuring the baseline of mental health. Previous studies demonstrate that childhood psychological problems have a long‐term effect on adult family income and other noneconomic outcomes (Goodman et al., 2011). Likewise, those with mental health issues or low self‐esteem may have difficulties forming and maintaining healthy relationships. Physical health and health behaviors learned in childhood may also have lasting effects on both adult mental health and the likelihood of marrying and staying married (Carr & Springer, 2010). Values—for example, religiosity, parental respect, and opinions about sex and marriage—may influence future decisions about marriage (Berrington & Diamond, 2000) and possibly adult mental health. Hence, examining the contribution of childhood experiences, values, and attitudes on future mental health helps us to better understand the relationship between partnership formation and mental well‐being.

## Method

**Figure 1:**
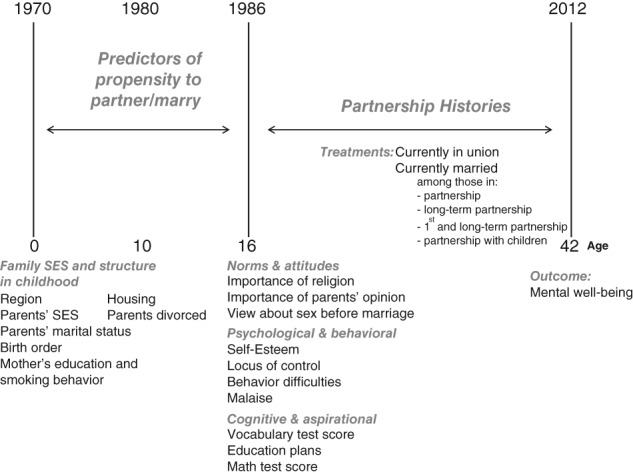
Methodological Scheme Based on the British Cohort Survey 1970. *Note*. SES = socioeconomic status.

To examine the effect of different partnership experiences on well‐being in midlife, we employed the British Cohort Study 1970 (BCS70), which is a nationally representative prospective survey of men and women born in Britain in 1 week of April 1970. We used data from sweeps 1970, 1980, 1986, and 2012 (http://www.cls.ioe.ac.uk/page.aspx?&sitesectionid=795&sitesectiontitle=Welcome+to+the+1970+British+Cohort+Study). Although attrition occurred throughout the period of data collection, follow‐up for the 2012 wave was relatively high, with a survey response rate of 75% (TNS BMRB, 2013). The cohort members were followed throughout their lives until age 42 (see Figure 1). Before the children reached adulthood, information about cohort members was provided by parents, teachers, nurses, and through self‐completed questionnaires. We restricted our sample to the men and women who participated in the survey at age 42 in 2012 (*N* = 9,841), who had valid well‐being scores (*n* = 8,070), were included in the survey since birth (and not added in subsequent booster samples; *n* = 7,471), and had valid partnership histories (*n* = 7,286). Finally, we eliminated those in same‐sex couples, because until recently they were unable to legally marry in the United Kingdom. This resulted in a final analytical sample of 7,203.

Figure 1 shows the waves of data collection in the BCS70 relevant to our methodological approach. The figure shows the years and age at which background characteristics, partnership trajectories, and the outcome variable were measured. The information on background characteristics was collected before partnership histories started, ensuring the correct causal ordering between background factors and the propensity to be in different relationship types. Partnership histories were collected starting in the 2000 wave and covered the period from when the cohort member was 16 to age 42. The Centre for Longitudinal Data provided a data file merging cleaned histories available up to 2008 (Hancock, 2011), and we updated partnership histories with the 2012 wave.

One of the limitations of this study is the proportion of missing responses, especially on some of the questions collected at age 16. Although the overall response rate for those who had been traced was around 88%, a teacher strike in 1986 meant about half of the students did not complete the in‐school questionnaires, which included the psychological attribute questions. The teacher strike was not restricted to particular locations in the United Kingdom, and the proportion of students attending each school sector was broadly representative (Gerova, 2006); therefore, the missingness in that wave seems to have occurred at random. However, nonresponse across the survey because of attrition did not seem to have occurred at random (Mostafa & Wiggins, 2014). Of those interviewed at age 42, about 5% of the original sample died, and 43% were missing as a result of emigration, noncontact, or refusal.

To preserve the sample size and reduce bias in the estimation effects because of the missing covariate values, we employed multiple imputation (MI) using chained equations (Rubin, 2004). Other analyses of the BCS70 concluded that MI was most effective at reducing bias as long as the MI was appropriately specified (Mostafa & Wiggins, 2014). Our imputation model included all covariates used for matching (see Table 1) as well as the well‐being score, partnership indicators, and having shared children. We also used a few auxiliary variables: Ever been in a partnership, ever married, reading test score, Rutter behavior score, self‐esteem score, and locus of control score were all measured at age 10. Using STATA 13 (StataCorp, 2013), we created 10 imputations separately for men and women. Scores measured at age 10 seemed to be very important for the imputation of scores measured at age 16, and because we wanted to include characteristics as close to entering adulthood as possible, we decided to use the imputed scores measured at age 16 and supplement them with other variables that captured norms and attitudes. Given the range of variables that we included in the MI and the similarity across models, we think that this approach sufficiently reduced the bias of nonresponse.

**Table 1:** Descriptive Statistics of Variables Included in the PSM Models

Variable		Frequency	Mean/percent
Family background indicators			
Region of birth	Scotland, Northern Ireland and North	2650	36.7
	Midlands and Wales	1631	22.7
	South West	506	7.1
	South East and East	2416	33.5
Social class at birth	V + IV unskilled and partly‐skilled	1433	19.9
	III manual	3145	43.7
	III non manual	1092	15.2
	I + II managerial, technical, professional	1525	21.2
Parent's marital status and timing of birth	not married	384	5.4
	married prior to conception	6153	86.8
	married after conception	551	7.8
Age mother finished education	14 years old or younger	428	6.0
	15 or 16 years old	5295	74.0
	17 years old or older	1435	20.0
Age of mother at first birth	<20	1610	22.5
	20‐24	3665	51.2
	25 and over	1885	26.3
Smoking behavior of mother during pregnancy	non smoker	3154	44.0
	stopped smoking before or during pregnancy	1280	17.9
	smoked during pregnancy	2734	38.1
Respondent's birth order	first child	2573	35.8
	second or next child	4624	64.2
Parent's place of birth	both parents born in the UK	6356	90.0
	at least one parent born outside the UK	710	10.0
Living with biological parents at age of 10	no	939	14.7
	yes	5459	85.3
Housing tenure (age 10)	owner occupier	4289	67.4
	public rented	1681	26.4
	other	391	6.2
Educational aspirations and cognitive development			
Respondent's plan to continue education	no	1766	53.4
	yes	1115	33.7
	don't know	425	12.9
Math test score at age 10	Math test score	5790	45.5 (11.8)
Vocabulary Test score at age 16	Standardized Vocabulary Test score	2772	0.1 (1.0)
Psychological measures			
Rutter behavior score	Index of behavior difficulties, derived using 19‐item Rutter Behavior Scale questions	4132	4.1 (3.8)
LAWSEQ	Scale of self‐esteem with reference to teachers, peers and parents and consisted of 10 items	2799	15.2 (3.4)
CARALOC	Locus of control scale, measures children's perceived achievement control, consisting of 19 items	2632	9.9 (3.0)
Malaise score	Scale to measure signs of psychological distress, based on Malaise Inventory	3048	9.0 (5.3)
Norms and attitudes			
Importance of religion in parental home	no religion	303	9.5
	not important	1874	59.0
	quite important	726	22.8
	very important	276	8.7
Importance of parental opinion	opinion of both parents important	3019	90.4
	opinion of only one parent important	218	6.5
	opinion of none of the parents important	104	3.1
Nothing wrong with sex before marriage	agree fully	1842	60.7
	agree partly	898	29.6
	disagree	295	9.7

### Estimating Union Formation Treatment Effects

To minimize the potential bias of nonrandom selection into different types of unions (the treatment), we used PSM and compared people with similar childhood background characteristics. In contrast to ordinary least squares regression, PSM allowed us to examine the heterogeneity of treatment effects based on whether people were more or less likely to marry. Propensity scores are conditional probabilities of experiencing the partnership treatment using logit regression (Guo & Fraser, 2014). To estimate the propensity score, we drew on factors and characteristics measured at birth in 1970, at age 10 in 1980, and age 16 in 1986. We then used PSM to match respondents in each sample based on the predicted probability to experience a given partnership status (the treatment). PSM models estimated the average effect of treatment among people who were similar by comparing well‐being scores (the outcome) among treated (average effect of the treatment on the treated) and untreated individuals (average effect of the treatment on the untreated). Nonetheless, PSM could not match people on unobservables, such as parents' marital quality in childhood, developments after childhood, and current relationship quality; therefore, it is unlikely to demonstrate a true causal effect. Next we show results that use kernel matching; nearest neighbor matching resulted in similar estimates. The covariates were unbalanced before matching, and matching effectively reduced the bias. For each covariate, the average mean bias after matching for all imputations was always below 5% (further information available on request).

#### Outcome variable

The Warwick‐Edinburgh Mental Well‐Being (WEMWB) Scale is a battery of 14 questions focusing on positive mental health, such as whether a respondent felt optimistic, loved, relaxed, confident, good about themselves, and so on (Tennant et al., 2007). Previous analysis has shown that the scale has good content validity and reliability in the United Kingdom (Tennant et al., 2007). The WEMWB score ranged from 14 to 70, and the mean was around 49. In our sample, it had a Cronbach's α of .92, indicating that it had high internal consistency. The score was only calculated if all 14 items were completed; it was available for 82% of respondents participating in the 2012 sweep. We used logistic regression to examine the characteristics associated with nonresponse of the WEMWB score and found that men, respondents with children, the unmarried, lower educated, and disabled were less likely to have a complete well‐being score, which could potentially underestimate raw differences between cohabitation and marriage, but should not influence the PSM results.

#### Treatments

Propensity score matching requires a binary treatment, which makes it difficult to analyze relationship duration or complex partnership transitions (i.e., cohabitation transitioning to marriage, or relationships that dissolve). Our solution was to present increasingly select groups to compare people with more similar unions (Table 1). We started by including everyone in the survey and examining whether being in a partnership mattered for well‐being (Sample 1). We then examined increasingly selective, or committed, types of unions and compared marriage with cohabitation (Samples 2–7). In the end, we compared people who were in relationships more similar to traditional marriage: first long‐term unions with children. The specific samples were the following:

1. Currently in a coresidential partnership (3,384 men and 3,819 women). This model demonstrated whether currently being in a relationship benefits mental well‐being for the entire survey sample. Those who were not in a partnership included the never partnered and the divorced or separated.
2. Currently married, among those currently in a partnership (2,620 men and 2,905 women). Model 2 was restricted to those currently in a partnership and examined whether currently being married boosts well‐being more than currently cohabiting.
3. Currently married, among those in a partnership lasting longer than 3 years (2,417 men and 2,684 women). This model examined differences between marriage and cohabitation only for those in a long‐lasting partnership. Anyone who was married in a coresidential partnership lasting longer than 3 years at time of interview was considered married, regardless of when the marriage occurred (i.e., they could have been married for fewer than 3 years, but premaritally cohabiting with the same partner for part of that time so that the total time spent together was more than 3 years). This approach minimized the “honeymoon effect” of relationship formation; previous studies found that forming a relationship in the last 2 to 3 years provides a boost to well‐being, but the initial gains subsequently diminish (Musick & Bumpass, 2012; Zimmerman & Easterlin, 2006). Note that this specification did not completely eliminate differences in the duration of cohabiting and marital unions, and on average, marital unions were longer than cohabiting unions.
4. Currently married, among those in a first relationship lasting longer than 3 years (1,746 men and 1,874 women). This sample only included men and women in long‐lasting relationships who had never experienced partnership dissolution. Note that we did not know whether the respondent's partner was also in a first relationship.

5–7.Samples 2, 3, and 4 for couples who havehad children together (respectively, 1,977,1,929, 1,465 men and 2,187, 2,134, 1,603women). We excluded childless couples andcouples who only had stepchildren, fosterchildren, or adopted children.

#### Background variables

We employed a number of strategies to select the covariates used to create the propensity score and settled on a model that included as many factors as possible. The variables represented a range of different domains: family background variables including parental socioeconomic status and divorce behavior, cognitive scores and educational aspirations, adolescent mental health and psychological attributes, and values such as religiosity and whether the respondent believed sex before marriage was wrong (Table 1). We tested the models with specific variables and sets of variables according to domain, but none had a particularly strong effect with the exception of psychological attributes for men. Thus, we present models with all childhood factors included.

## Results

### Descriptive Statistics

In Table 2 we report mean values, the standard deviations, and confidence intervals of the mental well‐being indicator (Warwick‐Edinburgh Mental Well‐Being Scale) by gender and treatment. About 23% of men and 24% of women lived outside of a partnership at the time of interview; those not in a partnership had significantly lower mean well‐being scores (46 for men and 47 for women) than those in a partnership (50 for men and women). Of those currently in a partnership, cohabitors had significantly lower mean well‐being scores than married people, but the difference was not as great as between the unpartnered versus partnered. The raw differences between those in long‐lasting cohabiting and marital relationships were also significant, but not as large, especially when people were in their first relationships. Differences were also small when they had children together. Nonetheless, cohabitors always had significantly lower well‐being scores than married people.

**Table 2:** Mean Mental Well‐Being Scores by Partnership Status

Category		Frequency	Percentage	Mean well‐being	*SD* of well‐being	95% CI
Men						
1.	Currently in partnership	2,620	77.4	49.9	7.7	[49.6, 50.2]
	Currently not in partnership	764	22.6	46.0	9.2	[45.4, 46.7]
2.	Currently married	2,073	79.1	50.2	7.5	[49.8, 50.5]
	Currently cohabiting	547	20.9	48.9	8.0	[48.3, 49.6]
3.	Long‐lasting marriage	2,011	83.2	50.1	7.5	[49.8, 50.4]
	Long‐lasting cohabitation	406	16.8	48.5	8.1	[47.7, 49.3]
4.	First and long‐lasting marriage	1,491	85.4	50.1	7.4	[49.7, 50.4]
	First and long‐lasting cohabitation	255	14.6	48.4	7.9	[47.4, 49.3]
5.	Currently married with own child(ren)	1,708	86.4	50.0	7.4	[49.7, 50.4]
	Currently cohabiting with own child(ren)	269	13.6	48.8	8.1	[47.8, 49.7]
6.	Long‐lasting marriage with own child(ren)	1685	87.4	50.0	7.4	[49.7, 50.4]
	Long‐lasting cohabitation with own child(ren)	244	12.6	48.6	7.9	[47.6, 49.6]
7.	First and long‐lasting marriage with own child(ren)	1,292	88.2	50.1	7.3	[49.7, 50.5]
	First and long‐lasting cohab. with own child(ren)	173	11.8	48.4	7.5	[47.3, 49.5]
Women						
1.	Currently in partnership	2,905	76.1	50.0	8.2	[49.7, 50.3]
	Currently not in partnership	914	23.9	46.8	9.0	[46.2, 47.4]
2.	Currently married	2,315	79.7	50.3	8.1	[50.0, 50.6]
	Currently cohabiting	590	20.3	48.6	8.4	[48.0, 49.3]
3.	Long‐lasting marriage	2,252	83.9	50.3	8.1	[50.0, 50.6]
	Long‐lasting cohabitation	432	16.1	48.4	8.4	[47.6, 49.1]
4.	First and long‐lasting marriage	1,613	86.1	50.4	8.2	[50.0, 50.8]
	First and long‐lasting cohabitation	261	13.9	48.2	8.0	[47.2, 49.1]
5.	Currently married with own child(ren)	1,926	88.1	50.3	8.2	[49.9, 50.7]
	Currently cohabiting with own child(ren)	261	11.9	48.4	8.4	[47.3, 49.4]
6.	Long‐lasting marriage with own child(ren)	1,896	88.8	50.3	8.2	[49.9, 50.6]
	Long‐lasting cohabitation with own child(ren)	238	11.2	48.5	8.4	[47.4, 49.5]
7.	First and long‐lasting marriage with own child(ren)	1,437	89.6	50.3	8.2	[49.9, 50.7]
	First and long‐lasting cohab. with own child(ren)	166	10.4	48.5	8.5	[47.2, 49.8]

*Source*. Author calculations with British Cohort Study 1970 data.

*Note*. CI = confidence interval; SD = standard deviation.

### Propensity Score Matching

In Table 3 we report mean differences between the Warwick‐Edinburgh Mental Well‐Being Scale scores and sample sizes for the treated and untreated groups by gender. Column 1 shows the number of treated and untreated used to calculate the unmatched differences presented in Column 3. The unmatched differences were statistically significant, in line with the descriptives from Table 2. Column 2 presents the number of treated and untreated cases restricted to the common support by the PSM procedure, which was used to calculate the average effects of the treatment on the treated (ATT) and average effects of the treatment on the untreated (ATU). Column 4 shows the ATT for those who were more likely to marry. Column 5 shows the ATU for those who were less likely to marry—often those targeted by marriage policies.

**Table 3:** Matching Estimates of Partnership Status on Mental Well‐being Scores at Age 42

		*N* entire sample	*N* under common support^a^	Unmatched difference	ATT	ATU
Men						
1.	T: Currently in partnership	2,620	2,606	3.88***	2.98***	2.88***
	C: Currently not in partnership	764	762	(0.33)	(0.45)	(0.40)
2.	T: Currently married	2,073	2,059	1.22**	0.64	0.54
	C: Currently cohabiting	547	545	(0.37)	(0.48)	(0.43)
3.	T: Long‐lasting marriage	2,011	1,997	1.65***	1.03	0.93
	C: Long‐lasting cohabitation	406	405	(0.41)	(0.54)	(0.51)
4.	T: First and long‐lasting marriage	1,491	1,475	1.71***	1.00	1.17
	C: First and long‐lasting cohabitation	255	254	(0.50)	(0.68)	(0.59)
5.	T: Currently married with own child(ren)	1,708	1,684	1.29*	0.32	0.59
	C: Currently cohabiting with own child(ren)	269	267	(0.49)	(0.70)	(0.59)
6.	T: Long‐lasting marriage with own child(ren)	1,685	1,658	1.47**	0.47	0.78
	C: Long‐lasting cohabitation with own child(ren)	244	242	(0.51)	(0.73)	(0.61)
7.	T: First and long‐lasting marriage with own child(ren)	1,292	1,265	1.64**	0.70	1.13
	C: First and long‐lasting cohab. with own child(ren)	173	170	(0.59)	(0.84)	(0.66)
Women						
1.	T: Currently in partnership	2,905	2,891	3.15***	2.26***	2.31***
	C: Currently not in partnership	914	912	(0.32)	(0.38)	(0.37)
2.	T: Currently married	2,315	2,304	1.66***	0.93*	1.03*
	C: Currently cohabiting	590	588	(0.38)	(0.46)	(0.41)
3.	T: Long‐lasting marriage	2,252	2,226	1.94***	0.97	1.26**
	C: Long‐lasting cohabitation	432	430	(0.43)	(0.54)	(0.47)
4.	T: First and long‐lasting marriage	1,613	1,603	2.20***	0.87	1.39*
	C: First and long‐lasting cohabitation	261	260	(0.54)	(0.73)	(0.63)
5.	T: Currently married with own child(ren)	1,926	1,891	1.93***	0.65	1.30*
	C: Currently cohabiting with own child(ren)	261	260	(0.54)	(0.72)	(0.58)
6.	T: Long‐lasting marriage with own child(ren)	1,896	1,838	1.81**	0.55	1.17
	C: Long‐lasting cohabitation with own child(ren)	238	237	(0.57)	(0.70)	(0.60)
7.	T: First and long‐lasting marriage with own child(ren)	1,437	1,424	1.78**	0.03	1.11
	C: First and long‐lasting cohab. with own child(ren)	166	164	(0.68)	(0.97)	(0.76)

*Note*. T refers to the treatment; C refers to the control. Numbers in parentheses are standard errors. Two‐tailed tests. ATT = average effects of the treatment on the treated; ATU = average effects of the treatment on the untreated.

a Calculated as mean over the imputations.

* *p* < .05;

** *p* < .01;

*** *p* < .001.

#### Unpartnered versus partnered

Both men and women who were in a cohabiting or marital partnership at interview had higher mental well‐being than those not in a coresidential partnership after taking childhood background characteristics into account (Model 1). This result was significant at the .001 level both for those more and less likely to be in a partnership. We found similar results after comparing the unpartnered with only those currently cohabiting (excluding the married): Those cohabiting had higher mental well‐being than the unpartnered, even after matching on childhood characteristics (significant at the .001 level). This indicates that the results were not primarily driven by the married (results available on request). Note that not being in a partnership may have reflected prior relationship break down (Hughes & Waite, 2002) or a long‐term decline in well‐being as a result of never partnering (Soons et al., 2009). However, unobserved factors that could currently impact well‐being, such as employment status or friendship networks, were not included in the models and may have further reduced and eliminated differences between the partnered and unpartnered. Indeed, Rosenbaum sensitivity checks indicated that a moderate amount of unobserved heterogeneity may have produced insignificant results. (The effect of unobserved heterogeneity is expressed as a factor [denoted as Γ] by which the odds of exposure to the treatment would change if unobserved variables played a role. A Γ of 1 implies no hidden bias. Values over 1 indicate that increasing levels of unobserved heterogeneity eventually bias the results. For men, our results might no longer be robust to unobserved heterogeneity for a Γ approximately greater than 2.5 for ATT and 1.7 for ATU. For women, the Γ was 1.8 for ATT and 1.5 for ATU.) Nonetheless, our results were robust to a number of different specifications of childhood characteristics that often predict future outcomes, suggesting that currently living with someone may provide a boost to well‐being.

#### Married versus cohabiting

After applying the propensity score matching, any significant differences in mental well‐being between married and cohabiting men disappeared completely for both ATU and ATT, regardless of union duration or having common children (Models 2–7). These findings indicated that for men, marriage did not have a significant effect on mental well‐being beyond simply living with someone; childhood selection effects completely explained the association. Thus, the British men born in 1970 did not appear to receive a positive benefit from marriage when compared with cohabitation.

For women, the results were less consistent: The ATT and ATU indicated differences between women who were more and less likely to marry. For those who were more likely to marry (ATT), marriage increased mental well‐being more than cohabitation among women currently in a union, an effect significant at the .05 level (Model 2). This result may have been picking up poor mental well‐being among those who had short‐term unions or had recently separated, but note that the difference in mental well‐being scores was less than one point, which may not be meaningful. Once we compared the ATT for relationships that were more committed, for example, those lasting more than 3 years (Models 3 and 4) or including children (Models 5–7), the differences between cohabiting and married women disappeared completely. This indicates that once partnerships become more committed, that is, last longer or include children, the type of union did not matter for women who were more likely to marry anyhow.

For women less likely to marry (ATU), for example, those from disadvantaged backgrounds or with low self‐esteem and high psychological distress in adolescence, marriage did seem to make a slight difference in adult mental well‐being, except for those in long‐lasting relationships with children. Those currently married had significantly higher well‐being than those who were cohabiting, significant at the .05 level (Model 2). Women in long‐lasting partnerships (Model 3) or even first partnerships (Model 4) also seemed to benefit from marriage, as did women who had children but may have been in shorter unions (Model 5). Only when we examined the most committed partnerships—those with shared children and lasting longer than 3 years (Models 6 and 7)—did we find that the well‐being of married and cohabitating women became similar. We tested numerous specifications of the models and found that the ATU for women without children remained significant at the .05 level. The richness of our indicators, all of which were measured before entrance into partnership, suggested that marriage did indeed convey benefits for women who were unlikely to marry. However, the PSM cannot rule out unobservables that selected women into cohabitation and resulted in worse mental well‐being (e.g., poor relationship skills or certain personality traits such as disagreeableness). Rosenbaum sensitivity analyses indicated that the results were sensitive to small amounts of unobserved heterogeneity. The analyses also did not control for mechanisms that would lead to positive mental health in adulthood, such as relationship quality, economic stability, or other support networks including friends or other relatives. Thus, although we observed significant differences for women who were less likely to marry, we urge caution in interpreting this as a causal effect.

## Discussion

This study provided insights into the role of marriage and cohabitation, and relationships in general, on mental well‐being in midlife in the United Kingdom. Although previous studies have examined the transition into different unions—usually occurring in early adulthood—few have investigated how different types of relationships matter during prime childrearing ages. Given that cohabitation at these ages has been increasing—in our sample one fifth of those living with a partner at age 42 were cohabiting—it is important to understand the consequences of this living arrangement for mental health, especially for those less likely to marry.

First, our findings demonstrate the importance of currently being in a coresidential relationship for adult mental well‐being. Living with an intimate partner seems to boost well‐being, possibly by providing emotional support, social networks, sexual intimacy, companionship, and social meaning—all of which positively influence mental well‐being (Umberson et al., 2010). Although we could not control for unobservable factors in childhood (such as personality, attractiveness, or physical health) or current factors (such as employment status, disability, poor health behaviors, or values and opinions), which may impact well‐being, the results nonetheless show that a wide range of childhood background characteristics usually predicting entrance into a partnership or future mental well‐being could not eliminate differences. Hence, although it is impossible to claim a causal effect, our results suggest that coresidential partnerships may matter for mental well‐being.

The type of partnership seems to matter little, especially once selection is taken into account. As found in other studies (Soons et al., 2009), we see a strong positive association between marriage and well‐being before controlling for selection, but after matching based on childhood factors, differences between cohabitation and marriage largely disappear, indicating that childhood selection mechanisms are sufficient to eliminate differences by union type. Our results indicate that conditions in childhood, such as parental socioeconomic status, family structure, cognitive ability and educational aspirations, psychological characteristics, and values and attitudes, are influencing both entrance into partnership and future mental well‐being. Nonetheless, results differ by gender, and although these differences are not large, they still raise questions about the meaning of cohabitation and marriage. For men, cohabitation appears to provide the social support necessary to bolster well‐being, including companionship, intimacy, and caretaking (Musick & Bumpass, 2012; Ross, 1995).

The results for women are more complicated. Women who are more likely to marry benefit little from marriage, suggesting that their childhood situation attenuates the association between marriage and mental health. Women who are less likely to marry, however, seem to get a small boost from marriage, unless they have children in long‐lasting relationships. The benefits for these disadvantaged women may be because marriage usually provides a more secure and stable environment through legal protection (Barlow, 2014), greater social recognition of the relationship, and higher levels of commitment expressed through a public vow (Berrington et al., 2015), all of which could in turn provide greater well‐being. In addition, marriage may accord with women's expectations of an ideal life course, including the experience of a traditional white wedding (Berrington et al., 2015). Thus, the greater stability that marriage provides may be more important for people coming from insecure backgrounds than for those who had an advantaged upbringing and who would have had other factors positively influencing their mental well‐being. However, we urge caution because our models may not have completely captured unobserved heterogeneity. In particular, part of the marriage effect among disadvantaged women may be a result of relationship quality, which was not measured in the BCS70; higher quality relationships may lead both to marriage and positive mental health. Investments such as having children and staying together may signal that the relationship is of higher quality, not requiring the official act of marriage. Thus, the gains to marriage that men and women receive may be different, with men benefiting from the coresidential aspects, but women who are less likely to marry benefiting marginally from the institutionalized nature of marriage that would provide them with a greater sense of well‐being.

Note that this study has several limitations. First, although our prospective, longitudinal data set is ideally suited for examining the effects of partnership on future well‐being while controlling for prior background characteristics, the BCS70 suffers from some attrition. We performed multiple imputation, but this approach assumes that variables measured at birth and age 10 generally predict adolescent characteristics, which leaves little independent development throughout adolescence and may overestimate the effect of early life conditions. Second, the PSM analysis requires that we define a single treatment, which limits how much we can account for characteristics about the partners and the complexity of partnership histories. To get around this, we examine increasingly committed types of unions, but we still do not directly compare individuals who have experienced union dissolution or subsequent repartnering. In addition, we cannot control for unions of different duration. If subjective well‐being declines during the duration of the partnership (Soons et al., 2009), and marital unions are on average longer, then some of the cohabiting unions may still be experiencing greater well‐being benefits as a result of more recent partnership formation, and in the long‐run, marriage may still confer greater benefits to well‐being. Despite these limitations, however, the results provide insights into the meaning of partnerships in Britain.

In Britain, partnership status in midlife is changing: Only 38% of our sample of 42‐year‐olds reported being in a first, long‐lasting marriage with children, indicating that more than half of all adults live in a situation different from traditional, long‐term marriage. Given the context of high dissolution rates and relationship turnover, cohabitation is becoming acceptable as one of those alternative living arrangements, with 16% of all adults in cohabitation at age 42. Relatively few, however, are in first long‐lasting cohabiting unions and have children together—only 5% of the entire sample. This small percentage suggests that long‐term cohabiting unions with children are still somewhat rare and that marriage is still the prevailing norm. On one hand, our results show that cohabitation in midlife, regardless of its duration or whether it includes children, is highly selective of characteristics associated with poor future mental health. Cohabitation tends to be associated with instability and suited to those struggling with unemployment, temporary jobs, or general life uncertainty (Berrington & Diamond, 2000; Cherlin, 2009; Perelli‐Harris et al., 2010). On the other hand, our results show that the official act of marriage does not lead to long‐term benefits to mental well‐being, except for some women who have a lower propensity to marry. Thus, it is unlikely that encouraging people to marry will improve mental well‐being.

In conclusion, this study has demonstrated the importance of early childhood conditions for understanding the relationship between cohabitation, marriage, and mental well‐being. Although previous studies comparing outcomes between cohabitation and marriage have generally controlled for contemporaneous selection effects (e.g., Brown, 2000; Lamb et al., 2003; Marcussen, 2005) or unobserved heterogeneity (e.g., Musick & Bumpass, 2012; Soons et al., 2009), to our knowledge none has specifically examined how selection mechanisms dating back to childhood and before entrance into a union explain the differential effects of marriage. Our study provides further evidence that early childhood conditions are important for understanding later life well‐being (Elo, 2009; Kuh et al., 2004). Taken together, these background characteristics play a strong role in eliminating most differences between cohabitation and marriage. Nonetheless, they cannot always explain away all differences, as was seen with the unpartnered and women less likely to marry. Overall, however, the results suggest that to improve mental well‐being, policy makers should focus on reducing the adverse effects of disadvantage in childhood and improving mental well‐being in adolescence rather than legislating incentives to marry in adulthood.

## Note

The research leading to these results has received funding from the European Research Council under the European Union's Seventh Framework Programme (FP7/2007‐2013) and European Research Council Grant 263794 CHILDCOHAB. The 1970 British Birth Cohort (BCS70) is conducted by the Centre for Longitudinal Studies, Institute for Education, University of London. This research was also funded by the Economic and Social Research Council Centre for Population Change (Grant RES‐625‐28‐0001). Access to all data is provided by the UK Data Archive. We thank colleagues at the University of Southampton for helpful suggestions on the study.
